# Analysis of Three-Dimensional Scar Architecture and Conducting Channels by High-Resolution Contrast-Enhanced Cardiac Magnetic Resonance Imaging in Chagas Heart Disease

**DOI:** 10.1590/0037-8682-0688-2021

**Published:** 2022-10-24

**Authors:** João Bosco de Figueiredo Santos, Ilan Gottlieb, Eduardo Marinho Tassi, Gabriel Cordeiro Camargo, Jacob Atié, Sérgio Salles Xavier, Roberto Coury Pedrosa, Josep Brugada, Roberto Magalhães Saraiva

**Affiliations:** 1 Fundação Oswaldo Cruz, Instituto Nacional de Infectologia Evandro Chagas, Rio de Janeiro, RJ, Brasil.; 2 Casa de Saúde São José, Setor de Radiologia, Rio de Janeiro, RJ, Brasil.; 3 Universidade Federal do Rio de Janeiro, Hospital Universitário Clementino Fraga Filho, Rio de Janeiro, RJ, Brasil.; 4 Universitat de Barcelona, Hospital Clínic, Institut Clínic Cardiovascular, Barcelona, Spain.

**Keywords:** Cardiac magnetic resonance, Myocardial fibrosis, Image integration, Conducting channels, Chagas heart disease, Prognosis

## Abstract

**Background:**

We aimed to describe the morphology of the border zone of viable myocardium surrounded by scarring in patients with Chagas heart disease and study their association with clinical events.

**Methods:**

Adult patients with Chagas heart disease (n=22; 55% females; 65.5 years, SD 10.1) were included. Patients underwent high-resolution contrast-enhanced cardiac magnetic resonance using myocardial delayed enhancement with postprocessing analysis to identify the core scar area and border zone channels number, mass, and length. The association between border zone channel parameters and the combined end-point (cardiovascular mortality or internal cardiac defibrillator implantation) was tested by multivariable Cox proportional hazard regression analyses. The significance level was set at 0.05. Data are presented as the mean (standard deviation [SD]) or median (interquartile range).

**Results:**

A total of 44 border zone channels (1[1-3] per patient) were identified. The border zone channel mass per patient was 1.25 (0.48-4.39) g, and the extension in layers of the border zone channels per patient was 2.4 (1.0-4.25). Most border zone channels were identified in the midwall location. Six patients presented the studied end-point during a mean follow-up of 4.9 years (SD 1.6). Border zone channel extension in layers was associated with the studied end-point independent from left ventricular ejection fraction or fibrosis mass (HR=2.03; 95% CI 1.15-3.60).

**Conclusions:**

High-resolution contrast-enhanced cardiac magnetic resonance can identify border zone channels in patients with Chagas heart disease. Moreover, border zone channel extension was independently associated with clinical events.

## INTRODUCTION

Chagas disease (CD) still affects six to seven million people worldwide. Although this neglected disease occurs mainly in Latin America, it is increasingly recognized as a new public health challenge in non-endemic countries due to migration^1^ ^,^ ^2^. Chronic Chagas heart disease (CHD) is characterized by chronic fibrosing myocarditis that leads to cardiac remodeling, heart failure (HF), arrhythmia, and cardioembolic stroke^3^ ^-^ ^5^.

Cardiac magnetic resonance imaging (MRI) permits the evaluation of the prognostic value of cardiac fibrosis in CHD. Indeed, cardiac fibrosis was reported to be associated with the occurrence of ventricular arrhythmias^6^ ^,^ ^7^, cardiovascular events^8^, and all-cause mortality^9^.

High-resolution contrast-enhanced cardiac MRI (ce-CMR) precisely differentiates the scar core and the morphology of the border zone (BZ) of viable myocardium surrounded by the scar core and/or the mitral annulus, and that connect two areas of normal myocardium^10^. These BZ channels have shown a close correlation with conducting channels on endocardial electroanatomic maps, allowing reentrant circuits^10^. In post-myocardial infarction patients, BZ size was associated with the inducibility of ventricular tachycardia (VT) and mortality^11^. BZ channel mass was an independent predictor of sustained VT in a retrospective case-control study among post-myocardial infarction patients^12^. Transmural BZ channels were also an independent predictor of post-ablation VT recurrence^13^. Furthermore, in ischemic and non-ischemic patients with left ventricular (LV) systolic dysfunction, BZ mass, and BZ channel mass were the strongest predictors of appropriate defibrillator therapy or sudden cardiac death^14^. In patients that implanted an internal cardiac defibrillator (ICD) for primary prevention, the identification of BZ channels or a scar mass larger than 10 g was associated with appropriate ICD therapy, in contrast with patients without those characteristics who presented a very low rate of appropriate therapies^15^.

Identifying BZ channels in CHD is important since sudden death is the primary mode of death in CHD^16^. ICD implantation is indicated as secondary prophylaxis in CHD for those who survive a sudden cardiac arrest or present sustained VT^17^. However, the clinical benefit of ICD in CHD is challenged by frequent appropriate shocks^18^ that VT ablation procedures could minimize. The mapping of BZ channels by ce-CMR may increase the success of such procedures in patients with CHD.

Therefore, our study aimed to evaluate if ce-CMR image postprocessing could identify BZ channels in patients with CHD and describe their morphology and association with clinical events.

## METHODS

### Study Subjects

This paper is the continuation of a previous project on the study of cardiac MRI in patients with CHD with and without ventricular arrythmias^6^. The current longitudinal and prospective study evaluated a convenience sample of adult patients of both sexes with chronic CHD who underwent ce-CMR for research purposes with a diagnosis of cardiac fibrosis between October 2014 and March 2016. All patients had regular appointments at the Clementino Fraga Filho University Hospital, Rio de Janeiro, Brazil, and had to be away from an area with endemic CD transmission for more than 20 years, present with chronic CHD according to the Brazilian CD consensus^3^, and test negative for ischemic heart disease on treadmill exercise testing. CD diagnosis was confirmed by positivity in two distinct serological tests^3^.

The exclusion criteria were as follows: clinical history of another concomitant heart disease, atrial flutter/fibrillation, thyroid disease, chronic kidney disease, two or more risk factors for coronary artery disease, pregnancy, and any contraindication to undergo cardiac MRI.

CHD classification criteria followed the Brazilian CD consensus^3^: stage A (electrocardiography [ECG] changes compatible with CHD but no HF symptoms or LV wall motion changes in echocardiography), stage B (asymptomatic segmental or global LV systolic dysfunction, subclassified into B1 or B2 according to LV ejection fraction higher or lower than 45%), stage C (history of HF), and stage D (end-stage HF).

Patients were clinically evaluated, had their epidemiological data collected, and underwent two-dimensional echocardiography, ECG, and treadmill exercise testing. Holter monitoring exams were completed for 14 patients at baseline. All exams for each patient were obtained within one month. After this evaluation was complete, patients were scheduled to undergo ce-CMR.

### Electrocardiogram

The 12-lead ECG tracing was interpreted following the Minnesota Code Manual of Electrocardiographic Findings (modified for CD)^19^. The ECG abnormalities that were compatible with CHD followed previously published criteria^3^.

### High-Resolution Ce-CMR

All ce-CMRs were acquired using a 3.0-T Siemens Verio (Siemens Healthcare, Erlangen, Germany) equipped with a cardiac 12-element phased-array coil and advanced cardiac-dedicated software. The images obtained were stored de-identified into DICOM format for offline analysis.

The LV images were acquired during a 15 s inspiratory breath-hold to decrease breathing movement artifacts. Longitudinal and short axis LV images were acquired by two ECG-triggered pulse sequences at the same spots. LV diameters, volumes, and ejection fraction, right ventricular (RV) volume and ejection fraction, and left atrial (LA) volume were evaluated by cine-CMR using the steady-state free precession protocol. LV mass was the result of the multiplication of LV myocardial volume by its myocardial tissue density (1.05 g/cm^3^). Images were acquired with 1.4 mm slice thickness with no space between slices up to the LV apex.

Images in the longitudinal and short axis projections were obtained using an inversion-recovery prepared gradient-echo for myocardial delayed enhancement (MDE) protocol 10 to 20 min after intravenous injection of 0.1 mmol/kg of gadolinium-based contrast (Dotarem^®^, Guerbet, France) to identify cardiac fibrosis. Fibrosis location and pattern were qualitatively determined. Fibrosis mass was calculated using a visual sub-segmental analysis as previously described^20^ and presented in absolute values and as a percentage of the LV mass. The LV 17-segment model was used for segmental MDE analysis^21^. Scar distribution patterns were classified into three categories as previously described^8^: transmural (any segment with fibrosis in more than 50% of its thickness, in up to eight segments); focal (no transmural scar in less than nine segments); and diffuse (scar areas identified in nine segments or more). Segmental fibrosis was categorized as transmural, subendocardial, midwall, or subepicardial.

### Ce-CMR Image Postprocessing

The postprocessing ce-CMR analyses were performed in Spain, as previously described^10^. Briefly, Automatic Detection of Arrhythmic Substrate - Ventricular Tachycardia (ADAS-VT) software was used in the Gimias environment (http://www.gimias.org). The LV epicardial and endocardial borders were automatically traced using three anatomic landmarks (mitral and aortic rings and the LV apex) and manually corrected as needed.

**FIGURE 1:**
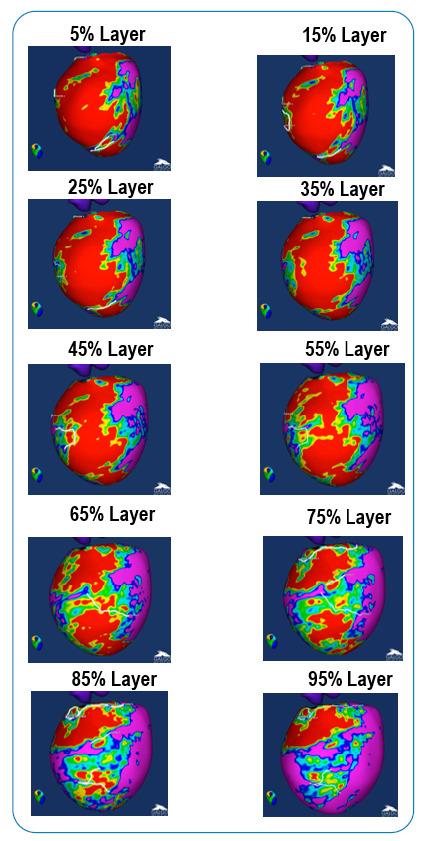
3-dimensional signal intensity maps in 10 out of 20 surface layers (from 5% to 95% of the LV wall thickness) obtained from a representative case depicting the normal myocardium (coded in purple), scar core (coded in red), and border zone (coded in blue-green-yellow).

Twenty concentric surface layers (from 5% to 95% of the LV wall thickness) were created through the myocardial wall from the endocardium to the epicardium (Figure 1). A 3D shell was obtained for each layer. The signal intensity (SI) distribution in each shell obtained from the ce-CMR was depicted by means of a color code in which the scar core areas were coded in red and BZ areas were coded in blue-green-yellow, using an algorithm based on maximum pixel SI in the LV wall. The automatic algorithm used a SI threshold above 60% of the maximum pixel SI to identify the core and from 40 to 60% of the maximum pixel SI to identify the BZ^22^. The extent and shape of the scar, categorized as core or BZ, was represented in three dimensions (3D) across the LV wall by the color-coded SI maps of the 20 transmural shells using the threshold-based classification.

**FIGURE 2:**
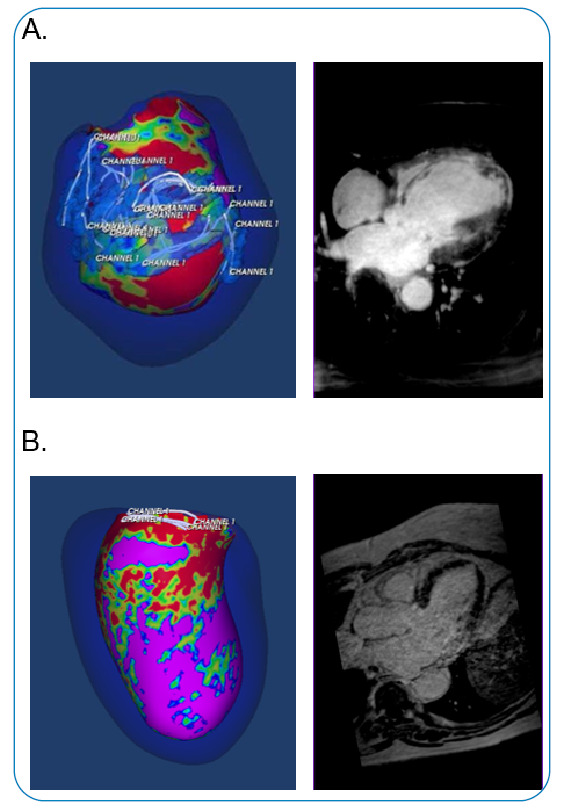
Signal intensity maps acquired from contrast-enhanced cardiac magnetic resonance representing in color-coded shells the left ventricle of a patient with a scar core area surrounded by a border zone (BZ) area with several long BZ channels **(A)** and another patient with a scar core area with a lower number of BZ channels with shorter extension **(B)**. Myocardial delayed enhancement-depicted areas of cardiac fibrosis in all segments of the septum, apex, and apical segment of the inferior wall in **A** and in the basal and mid segments of the inferolateral wall in **B.** The signal intensity codification is as follows, normal myocardium: purple, scar core: red, and BZ: blue-green-yellow. The BZ in light blue contains the BZ channels coded in white.

The software enables the 3D identification of the core, BZ channel number, mass, location, and extension using a specific function to calculate the geodesic distance between two given points in a shell. The BZ was coded light blue, with the BZ channels coded white (Figure 2).

### Study End-Point and Follow-up

The study end-point was a composite of cardiovascular death or ICD implantation. Patients were regularly followed up at Clementino Fraga Filho University Hospital during the study follow-up, and phone contact was made if a patient missed their clinical appointments. Additional Holter monitoring exams were performed whenever patients developed new symptoms of syncope or near syncope. Amiodarone was prescribed for all patients with: 1 - LV ejection fraction under 40% and non-sustained VT on Holter, and history of syncope or sustained VT or ventricular fibrillation (VF) inducible during electrophysiological study; 2 - sustained VT/VF associated with symptoms or not. Electrophysiological studies were performed in case of ICD implantation indication or the presence of non-sustained VT and LV ejection fraction under 40%. ICD implantation was indicated for all patients with either: 1-sudden cardiac arrest due to sustained VT/VF and LV ejection fraction under 35%; or 2-sustained VT and LV ejection fraction under 35%; or 3-non-sustained VT and LV ejection fraction under 35% and sustained VT/VF inducible during electrophysiological study.

Death was classified as sudden if a previously stable patient died within 1 h of symptom onset, during sleep, or unwitnessed. Acute non-cardiac illness, drug overdose, terminal disease, metabolic causes, and vascular non-cardiac disease should be ruled out before the etiology was considered cardiac^23^. Information about death was obtained from institutional medical records and from a direct interview with participants’ relatives, who were also requested to provide copies of the death certificates.

### Statistical Analysis

Calculations were performed using MedCalc 12.5.0.0 statistical software. Normally distributed continuous variables are expressed as the mean and standard deviation (SD), while skewed distributed variables are expressed as the median (interquartile range). Kolmogorov-Smirnov tests demonstrated that the continuous variables were normally distributed if the obtained P-values were >0.10. Categorical variables are expressed as absolute and percentage values. Comparisons between groups were performed using an unpaired Student’s *t-*test or Mann-Whitney test, as appropriate. Separate multivariable Cox proportional hazards regression analyses adjusting for LV ejection fraction and LV fibrosis mass were performed to test if the BZ channel parameters were associated with the studied end-point. LV ejection fraction^24^ and fibrosis mass^9^ were chosen as the adjusting variables based on previous studies that demonstrated their value as mortality predictors in CD. Model Harrell’s C-index (Harrell)^25^ was calculated for each multivariate model and represents a goodness of fit measure for models. Values of C were interpreted as the area under the receiver operating characteristic curve. A value of C close to 0.5 indicated that the model was not superior to random chance to predict an outcome, while values > 0.7 indicated a good prediction model. The modified τ method was used to determine the confidence interval of Harrell’s C-index^26^. Schoenfeld residuals did not discard the proportional assumption of Cox proportional hazards regression analysis. The significance level was set at 0.05.

### Ethical Considerations

This study was approved by the institutional ethics committee of the Federal University of Rio de Janeiro (651/09; 05/04/2009 and 1.186.956; 08/16/2015), conforms to standards applied by the Brazilian National Committee for Research Ethics and Resolution 466/2012 of the National Health Council of the Ministry of Health, and complies with the Declaration of Helsinki, 1964, as revised in 1975, 1983, 1989, 1996, and 2000. All patients gave informed consent before participating.

## RESULTS

### Patient Characteristics

A total of 22 patients (55% females; 65.5 years (SD 10.1) were included in this study. Most of the patients were in the earlier stages of CHD (stage A or B1). Nine of these patients (40.9%) presented the associated digestive form. There was a similar sex distribution. Most patients were white, originated from the Northeast Brazilian region, and had arterial hypertension. The most common symptoms were palpitations, near syncope, and syncope (Table 1).

**TABLE 1:** Baseline epidemiological and clinical features of the participants (n=22).

Age (years)	65.5 (10.1)
**Sex**	
Males	10 (45)
Females	12 (55)
**Brazilian region**	
Northeast	15 (68.2)
Southeast	5 (22.7)
Central-West	2 (9.1)
**Race**	
White	15 (68.2)
Mixed/Pardo	5 (2.7)
Afro-Brazilian	2 (9.1)
**CHD clinical form**	
Cardiac stage A	8 (36.4)
Cardiac stage B1	11 (50)
Cardiac stage B2	1 (4.5)
Cardiac stage C	2 (9.1)
**Symptoms**	
Near syncope	5 (22.7)
Syncope	3 (13.6)
Palpitations	8 (36.4)
**HTN**	13 (59.1)
**Electrocardiogram**	
RBBB	
3^0^ degree	5 (22.7)
2^0^ degree	11 (50)
1^0^ degree	4 (18.2)
LAHB	16 (72.7)
Primary T wave changes	21 (95.4)
Premature ventricular complex	8 (36.4)
Inactive electrical zone	1 (4.5)
**Holter (n=14)**	
Premature ventricular complexes	
>30/h	7 (50)
10-30/h	6 (42.8)
<10/h	1 (7.1)
Non-sustained ventricular tachycardia	4 (28.6)
Sustained ventricular tachycardia	1 (7.1)
Sinus pauses >2 s	3 (21.4)
**Medications**	
Carvedilol	22 (100)
ACE inhibitor/ARB	14 (63.6)
Spironolactone	2 (0.9)
Amiodarone	3 (13.6)

**CHD:** Chagas heart disease; **HTN:** hypertension; **LAHB:** left anterior hemiblock; **LV:** left ventricular; **RBBB:** right bundle branch block. Values are shown as the mean and standard deviation or n (%).

The most common ECG abnormalities were primary T wave changes, right bundle branch block, left anterior hemiblock, and premature ventricular complex (Table 1). The most common abnormalities recorded on 24 h Holter monitors were premature ventricular complexes, followed by non-sustained VT and sinus pause >2 s. Only one participant had sustained VT (Table 1).

### Ce-CMR

The LV volumes, end-diastolic diameter and ejection fraction, LA volume, and RV end-diastolic volume and ejection fraction are depicted in Table 2. A total of 12 (54.5%) patients presented with LV systolic dysfunction, while only one presented with an RV ejection fraction below 45%. Among the patients with LV systolic dysfunction, five had mild, six had moderate, and one had severe, according to criteria previously published^27^.

**TABLE 2:** Cardiac structure and LV fibrosis characteristics on ce-CMR.

Variable	Total (n=22)	Without Event (n=16)	With Event (n=6)	P-value
End-diastolic LV diameter, mm	54.1 (7.7)	52.2 (7.3)	59.2 (7.0)	0.06
End-diastolic LV volume, mL/m^2^	80.3 (25.4)	75.4 (22.6)	93.5 (30.1)	0.14
End-systolic LV volume, mL/m^2^	42.0 (22.6)	37.4 (19.0)	54.2 (28.7)	0.12
LV ejection fraction, %	50.8 (12.5)	53.2 (12.1)	44.3 (12.1)	0.14
LV mass, g/m^2^	56.6 (12.1)	54.2 (11.2)	63.2 (12.7)	0.12
Left atrial volume, mL/m^2^	47.1 (17.6)	46.7 (14.8)	48.3 (25.4)	0.86
End-diastolic RV volume, mL/m^2^	61.0 (15.3)	63.6 (15.7)	54.3 (13.3)	0.22
RV ejection fraction, %	56.3 (9.9)	56.6 (11.6)	55.5 (3.3)	0.83
Fibrosis mass in grams	19.1 (15.7)	16.4 (13.6)	32.8 (13.5)	**0.009**
Fibrosis mass in % of LV mass	20.1 (14.8)	14.0 (13.5)	30.2 (14.0)	**0.048**
BZ channel mass in grams	1.2 (0.5-4.4)	0.7 (0.5-1.7)	5.4 (2.1-13.9)	**0.01**
Number of BZ channels	1.0 (1.0-3.0)	1.0 (1.0-2.0)	2.5 (1.0-5.5)	0.16
BZ channels extension in layers	2.4 (1.0-4.25)	1.2 (1.0-2.95)	5.1 (3.6-7.0)	**0.006**

**BZ:** border zone; **LV:** left ventricular; **RV:** right ventricular. Values are shown as the mean and standard deviation or median (interquartile range).

The fibrosis pattern was focal in 13 patients (59.1%), transmural in four (18.2%), and diffuse in five (22.7%). The number of walls with areas of fibrosis was 117 of 374 (31.3%), while the percentage of the walls presenting fibrosis areas was as follows: basal anteroseptal (31.8%), basal anterior (18.2%), basal anterolateral (36.4%), basal inferolateral (77.3%), basal inferior (45.4%), basal inferoseptal (18.2%), mid anteroseptal (18.2%), mid anterior (18.2%), mid anterolateral (36.4%), mid inferolateral (40.9%), mid inferior (18.2%), mid inferoseptal (22.7%), apical septal (27.3%), apical anterior (27.3%), apical lateral (36,4%), apical inferior (27.3%), and apex (31.8%). The segmental fibrosis pattern was midwall in 82 segments, transmural in 32, subendocardial to midwall in two, and subepicardial to midwall in one.

A total of 44 BZ channels were identified (Table 2). One patient presented with seven BZ channels, three with four BZ channels, two with three BZ channels, three with two BZ channels, and 13 with one BZ channel. Regarding CHD stages, one patient with stage C disease had seven channels, while another had one channel; the patient with stage B2 disease had four channels; those with stage B1 disease had 2.1 channels (SD 1.2), and those with stage A disease had 1.1 channels (SD 0.3). Twenty-one (47.7%) BZ channels were confined to just one layer; five (11.4%) to two layers; five (11.4%) to three layers; four (9.1%) to four layers; one BZ channel (2.3%) each was confined to five, six, seven, eight, 10, 13, 15, 16, and 18 contiguous layers. Most BZ channels were identified in the midwall (n=28; 63.6%), followed by the subendocardium (n=6; 13.6%) and subepicardium (n=2; 4.5%). The other four BZ channels (9.1%) had a path through all layers of the myocardium (transmural), two BZ channels (4.5%) showed a path involving the midwall and the subendocardium, and two BZ channels (4.5%) showed a path involving the midwall and the subepicardium.

### Study End-Point and Survival Analysis

Six patients presented the studied combined end-point during a mean follow-up of 4.9 years (SD 1.6). Five patients had cardiac sudden death, and two underwent ICD implantation due to inducible sustained VT during an electrophysiological study that was performed due to non-sustained VT and LV ejection fraction under 40%. One of the patients who underwent ICD implantation died 22 months after placement. Those patients who presented the studied end-point during the study follow-up had larger LV fibrosis mass, larger BZ channel mass, and higher BZ channel extension in layers at baseline (Table 2).

The results of the univariable analyses are depicted in Tables 3, 4, and 5. LV fibrosis mass was associated with the studied end-point in the univariable analysis. Age (HR=0.94; 95% CI 0.85-1.04) and sex (HR=2.8; 95% CI 0.5-15.3) were not included as adjustment variables of the multivariable models.

**TABLE 3:** Multivariable model assessing the value of BZ channel mass for predicting cardiovascular events.

	Univariable analysis			Multivariable analysis			Model Harrell’s C-index
Variable of interest	HR	95% CI	P-value	HR	95% CI	P-value	
LV ejection fraction, %	0.95	0.89-1.02	0.17	0.98	0.88-1.09	0.71	0.77 (0.63-0.91)
Fibrosis mass in grams	1.05	1.01-1.09	0.02	1.10	0.99-1.22	0.07	
BZ channel mass, g	1.09	0.99-1.22	0.09	0.86	0.65-1.13	0.28	

**BZ:** border zone.

**TABLE 4:** Multivariable model assessing the value of the number of border zone channels for predicting cardiovascular events.

	Univariable analysis			Multivariable analysis			Model Harrell’s C-index
Variable of interest	HR	95% CI	P-value	HR	95% CI	P-value	
LV ejection fraction, %	0.95	0.89-1.02	0.17	1.00	0.88-1.12	0.99	0.80 (0.71-0.90)
Fibrosis mass in grams	1.05	1.01-1.09	0.02	1.05	0.99-1.11	0.11	
Number of BZ channels	1.38	0.96-2.00	0.08	0.97	0.46-2.02	0.93	

**BZ:** border zone.

**TABLE 5:** Multivariable model assessing the value of the BZ channels extension in layers for predicting cardiovascular events.

	Univariable analysis			Multivariable analysis			Model Harrell’s C-index
Variable of interest	HR	95% CI	P-value	HR	95% CI	P-value	
LV ejection fraction, %	0.95	0.89-1.02	0.17	1.00	0.92-1.09	0.96	0.89 (0.80-0.98)
Fibrosis mass in grams	1.05	1.01-1.09	0.02	1.04	0.99-1.10	0.09	
BZ channels extension in layers	1.82	1.19-2.78	0.006	2.02	1.13-3.63	0.02	

**BZ:** border zone.

BZ channel extension in layers was associated with the studied end-point independent from LV ejection fraction or LV fibrosis mass, while other BZ channel characteristics (mass and number) had a borderline univariable association with the studied end-point. However, the BZ channel mass and number were not associated with the studied end-point after multivariable analysis (Tables 3, 4, and 5). LV fibrosis mass remained with a borderline association with the studied end-point after multivariable analyses. The average number of layers of the BZ channels of the patients that presented events were 3.3, 3.9, four, 6.25, and seven (two cases). No patient in whom all BZ channels had a single layer in extension presented a cardiac event.

## DISCUSSION

To our knowledge, this is the first study to evaluate BZ channels and cardiac fibrosis by postprocessing ce-CMR analyses using ADAS-VT software in patients with CHD. This software performs a 3D analysis of the scar and BZ, allowing the identification of the conducting channels corresponding to the electrophysiological substrate of the VT isthmus^10^. This information can be handy to the electrophysiologist during catheter ablation procedures by facilitating the identification of the ideal location for the ablation. Furthermore, the BZ channel extension in layers was associated with the occurrence of cardiovascular events independent of LV fibrosis mass and ejection fraction.

### Scar Architecture and BZ Channel

The architectural scar pattern in patients with coronary artery disease is pyramidal, with the subendocardial area being more extensive than the subepicardial area and a more significant number of BZ channels having a subendocardial location. Moreover, the BZ channels with a subepicardial location correspond to 35% of the BZ channels in transmural scar zones^10^. In our study, the fibrosis pattern of most patients was focal and midwall, as described previously in patients with earlier stages of CHD^28^. Although the BZ channel mass and number reported by us in CHD is within the range previously published in other cardiac diseases^14^ ^,^ ^15^, the BZ channel pattern differed in CHD as almost half of the BZ channels were confined to a single layer, and most were identified in the mesocardium rather than in a subendocardial location. This information is crucial for radiofrequency ablation planning.

### Clinical Implications

As sudden cardiac death (SCD) is the primary mode of death in CHD^16^, strategies to treat malignant ventricular arrhythmias may increase survival in these patients. This increase in survival can be even more significant in patients with earlier-stage CHD since they do not present with HF. Moreover, as the proportion of patients with earlier-stage CHD is higher than that of patients with CHD and HF, most episodes of SCD occur among the former^16^. ICD implantation would increase survival in CHD and is recommended for secondary prevention^17^. However, the long-term benefit of ICD use for primary or even secondary prevention remains controversial since no randomized controlled trial to date has tested ICD against pharmacological treatment. One of the reasons for this controversy is the high mortality that patients with CHD present despite ICD implantation^18^ ^,^ ^29^ ^-^ ^31^. However, there is great heterogeneity among mortality rates reported after ICD implantation, possibly due to discrepancies in study populations, concomitant use of amiodarone and carvedilol, and ICD programming characteristics. Nevertheless, Gali et al. compared patients treated with ICD plus amiodarone against a historical control of patients treated with only amiodarone for secondary VT prevention and described a decrease in 72% of the risk of all-cause mortality and of 95% in SCD among those treated with ICD implantation^32^. The knowledge gained with BZ and 3D scar characterization may aid in the identification of patients with higher SCD risk among those at earlier stages of CHD and change the balance toward an absolute survival benefit among patients selected for ICD implantation. Even in a small sample of patients, our results indicate that the BZ channel extension is a potential new risk factor for SCD in patients with CHD that deserves further studies in larger populations. Similarly, BZ channel extension, characterized by its transmural extension, was an independent predictor of post-ablation VT recurrence in patients with ischemic and non-ischemic cardiopathy, while BZ channel mass or number were not^13^. The extension of the BZ channel may be an important arrhythmic predictor as BZ conductive channels are a fundamental component of the reentrant circuit of ventricular arrhythmias. BZ channel mass was also associated with arrhythmic events in post-myocardial infarction patients^12^ and in patients with ischemic and non-ischemic cardiomyopathy^14^. Therefore, ce-CMR allows the analysis not only of the scar but also of the BZ conductive channels and may improve SCD prediction in CHD.

One fact that could contribute to the mortality of patients with CHD despite ICD implantation is the large number of inappropriate versus appropriate shocks. In fact, among patients with CHD and ICD for secondary prophylaxis, those who received more shocks within 30 days had higher cardiac mortality rates^18^. The mapping of BZ channels may aid in the identification of patients at higher risk of malignant arrhythmic events who could benefit from VT ablation. The mapping of BZ channels may also help plan VT ablation, as the ablation technique depends on whether it has to be done via an endocardial or epicardial approach.

### Limitations

The small sample of patients and clinical events included in the current study limit the conclusions. The small number of events precluded the inclusion of other relevant adjustment variables, such as age, sex, and medications used, and may cause overfitting. The small sample size also did not allow the necessary statistical power to imply conclusions over variables not associated with the combined outcome. The BZ mass was not analyzed in this study. This variable was described by others as a predictor of cardiovascular outcomes in other clinical settings^11^ ^,^ ^14^ and could have been included among the adjusting variables. Another limitation is that Holter monitoring exams were not obtained at baseline in all patients. However, additional Holter exams were obtained during follow-up whenever patients presented with symptoms of syncope or near syncope. Regardless, the findings of our survival analysis support the hypothesis that BZ channel architecture may provide additional predictive value to SCD prediction models.

## CONCLUSIONS

In this group of CHD patients, ce-CMR with ADAS-VT allowed the identification of BZ channels as well as their characteristics, precise location, and trajectory. Moreover, BZ channel extension was independently associated with the occurrence of clinical events. Our findings underscore the potential importance of BZ channel identification by ce-CMR for therapeutic guidance and identification of high-risk patients. Further studies are warranted in this area.

## References

[1] Gascon J, Bern C, Pinazo MJ (2010). Chagas disease in Spain, the United States and other non-endemic countries. Acta Trop.

[2] Mills RM (2020). Chagas Disease: Epidemiology and Barriers to Treatment. Am J Med.

[3] Dias JC, Ramos AN, Gontijo ED, Luquetti A, Shikanai-Yasuda MA, Coura JR (2016). 2 nd Brazilian Consensus on Chagas Disease, 2015. Rev Soc Bras Med Trop.

[4] Bonney KM, Luthringer DJ, Kim SA, Garg NJ, Engman DM (2019). Pathology and Pathogenesis of Chagas Heart Disease. Annu Rev Pathol.

[5] Rossi MA (1991). The pattern of myocardial fibrosis in chronic Chagas' heart disease. Int J Cardiol.

[6] Tassi EM, Continentino MA, Nascimento EM, Pereira BB, Pedrosa RC (2014). Relationship between fibrosis and ventricular arrhythmias in Chagas heart disease without ventricular dysfunction. Arq Bras Cardiol.

[7] Mello RP, Szarf G, Schvartzman PR, Nakano EM, Espinosa MM, Szejnfeld D (2012). Delayed enhancement cardiac magnetic resonance imaging can identify the risk for ventricular tachycardia in chronic Chagas' heart disease. Arq Bras Cardiol.

[8] Volpe GJ, Moreira HT, Trad HS, Wu KC, Braggion-Santos MF, Santos MK (2018). Left Ventricular Scar and Prognosis in Chronic Chagas Cardiomyopathy. J Am Coll Cardiol.

[9] Senra T, Ianni BM, Costa ACP, Mady C, Martinelli-Filho M, Kalil-Filho R (2018). Long-Term Prognostic Value of Myocardial Fibrosis in Patients With Chagas Cardiomyopathy. J Am Coll Cardiol.

[10] Fernandez-Armenta J, Berruezo A, Andreu D, Camara O, Silva E, Serra L (2013). Three-dimensional architecture of scar and conducting channels based on high resolution ce-CMR: insights for ventricular tachycardia ablation. Circ Arrhythm Electrophysiol.

[1] Yan AT, Shayne AJ, Brown KA, Gupta SN, Chan CW, Luu TM (2006). Characterization of the peri-infarct zone by contrast-enhanced cardiac magnetic resonance imaging is a powerful predictor of post-myocardial infarction mortality. Circulation.

[12] Jauregui B, Soto-Iglesias D, Penela D, Acosta J, Fernandez-Armenta J, Linhart M (2022). Cardiovascular magnetic resonance determinants of ventricular arrhythmic events after myocardial infarction. Europace.

[13] Quinto L, Sanchez P, Alarcon F, Garre P, Zaraket F, Prat-Gonzalez S (2021). Cardiac magnetic resonance to predict recurrences after ventricular tachycardia ablation: septal involvement, transmural channels, and left ventricular mass. Europace.

[14] Acosta J, Fernandez-Armenta J, Borras R, Anguera I, Bisbal F, Marti-Almor J (2018). Scar Characterization to Predict Life-Threatening Arrhythmic Events and Sudden Cardiac Death in Patients With Cardiac Resynchronization Therapy: The GAUDI-CRT Study. JACC Cardiovasc Imaging.

[15] Sanchez-Somonte P, Quinto L, Garre P, Zaraket F, Alarcon F, Borras R (2021). Scar channels in cardiac magnetic resonance to predict appropriate therapies in primary prevention. Heart Rhythm.

[16] de Souza AC, Salles G, Hasslocher-Moreno AM, de Sousa AS, Alvarenga Americano do Brasil PE, Saraiva RM (2015). Development of a risk score to predict sudden death in patients with Chaga's heart disease. Int J Cardiol.

[17] Andrade JP, Marin-Neto JA, Paola AA, Vilas-Boas F, Oliveira GM, Bacal F (2011). Latin American guidelines for the diagnosis and treatment of Chagas cardiomyopathy. Arq Bras Cardiol.

[18] Cardinalli-Neto A, Bestetti RB, Cordeiro JA, Rodrigues VC (2007). Predictors of all-cause mortality for patients with chronic Chagas' heart disease receiving implantable cardioverter defibrillator therapy. J Cardiovasc Electrophysiol.

[19] Maguire JH, Mott KE, Souza JA, Almeida EC, Ramos NB, Guimaraes AC (1982). Electrocardiographic classification and abbreviated lead system for population-based studies of Chagas' disease. Bull Pan Am Health Organ.

[20] Fine NM, Tandon S, Kim HW, Shah DJ, Thompson T, Drangova M (2013). Validation of sub-segmental visual scoring for the quantification of ischemic and nonischemic myocardial fibrosis using late gadolinium enhancement MRI. J Magn Reson Imaging.

[21] Cerqueira MD, Weissman NJ, Dilsizian V, Jacobs AK, Kaul S, Laskey WK (2002). Standardized myocardial segmentation and nomenclature for tomographic imaging of the heart. A statement for healthcare professionals from the Cardiac Imaging Committee of the Council on Clinical Cardiology of the American Heart Association. Circulation.

[22] Andreu D, Berruezo A, Ortiz-Perez JT, Silva E, Mont L, Borras R (2011). Integration of 3D electroanatomic maps and magnetic resonance scar characterization into the navigation system to guide ventricular tachycardia ablation. Circ Arrhythm Electrophysiol.

[23] Deyell MW, Krahn AD, Goldberger JJ (2015). Sudden cardiac death risk stratification. Circ Res.

[24] Rassi A, Rassi A, Rassi SG (2007). Predictors of mortality in chronic Chagas disease: a systematic review of observational studies. Circulation.

[25] Harrell FE, Lee KL, Mark DB (1996). Multivariable prognostic models: issues in developing models, evaluating assumptions and adequacy, and measuring and reducing errors. Stat Med.

[26] Pencina MJ, D'Agostino RB (2004). Overall C as a measure of discrimination in survival analysis: model specific population value and confidence interval estimation. Stat Med.

[27] Petersen SE, Khanji MY, Plein S, Lancellotti P, Bucciarelli-Ducci C (2019). European Association of Cardiovascular Imaging expert consensus paper: a comprehensive review of cardiovascular magnetic resonance normal values of cardiac chamber size and aortic root in adults and recommendations for grading severity. Eur Heart J Cardiovasc Imaging.

[28] Gomes VA, Alves GF, Hadlich M, Azevedo CF, Pereira IM, Santos CR (2016). Analysis of Regional Left Ventricular Strain in Patients with Chagas Disease and Normal Left Ventricular Systolic Function. J Am Soc Echocardiogr.

[29] Martinelli M, de Siqueira SF, Sternick EB, Rassi A, Costa R, Ramires JÁ (2012). Long-term follow-up of implantable cardioverter-defibrillator for secondary prevention in chagas' heart disease. Am J Cardiol.

[30] Pavao MLRC, Arfelli E, Scorzoni-Filho A, Rassi A, Pazin-Filho A, Pavao RB (2018). Long-term follow-up of Chagas heart disease patients receiving an implantable cardioverter-defibrillator for secondary prevention. Pacing Clin Electrophysiol.

[31] Di TD, Muratore C, Aguinaga L, Batista L, Malan A, Greco O (2011). Predictors of all-cause 1-year mortality in implantable cardioverter defibrillator patients with chronic Chagas' cardiomyopathy. Pacing Clin Electrophysiol.

[32] Gali WL, Sarabanda AV, Baggio JM, Ferreira LG, Gomes GG, Marin-Neto JÁ (2014). Implantable cardioverter-defibrillators for treatment of sustained ventricular arrhythmias in patients with Chagas' heart disease: comparison with a control group treated with amiodarone alone. Europace.

